# Comparing new treatments for idiopathic pulmonary fibrosis – a network meta-analysis

**DOI:** 10.1186/s12890-015-0034-y

**Published:** 2015-04-18

**Authors:** Emma Loveman, Vicky R Copley, David A Scott, Jill L Colquitt, Andrew J Clegg, Katherine MA O’Reilly

**Affiliations:** Effective Evidence LLP/Southampton Health Technology Assessments Centre (SHTAC), University of Southampton, 1st Floor Epsilon House, Enterprise Road, Southampton, SO16 7NS UK; SHTAC, University of Southampton, 1st Floor Epsilon House, Enterprise Road, Southampton, SO16 7NS UK; ICON Health Economics, Seacourt Tower, West Way, Oxford, OX2 0JJ UK; Mater Misericordiae University Hospital, Dublin, Ireland

**Keywords:** Idiopathic pulmonary fibrosis, Systematic review, Network meta-analysis

## Abstract

**Background:**

The treatment landscape for idiopathic pulmonary fibrosis, a devastating lung disease, is changing. To investigate the effectiveness of treatments for idiopathic pulmonary fibrosis we undertook a systematic review, network meta-analysis and indirect comparison.

**Methods:**

We searched MEDLINE, EMBASE and The Cochrane library for relevant studies. Randomised controlled trials of pirfenidone, nintedanib or N-acetylcysteine were eligible. Predefined processes for selecting references, extracting data and assessing study quality were applied. Our network meta-analysis of published data used a fixed effect model. For forced vital capacity measures a standardised mean difference approach was used and converted to odds ratios for interpretation.

**Results:**

Of 1076 references, 67 were retrieved and 11 studies included. Studies were of reasonable size, populations were similar, and the overall quality was good. Only two treatments, pirfenidone (odds ratio 0.62, 95% credible interval 0.52, 0.74) and nintedanib (0.41, 95% credible interval 0.34, 0.51) produced a statistically significant slowing in the rate of forced vital capacity decline compared with placebo. In an indirect comparison, results indicate that nintedanib is statistically significantly better than pirfenidone in slowing forced vital capacity decline (odds ratio 0.67, 95% credible interval 0.51, 0.88). Results were stable in scenario analysis and random effects models. Indirect comparisons of mortality were not statistically significant between nintedanib and pirfenidone.

**Conclusions:**

Two treatments show beneficial effects and when compared indirectly nintedanib appears to have superior benefit on forced vital capacity. Limitations to indirect comparisons should be considered when interpreting these results, however, our findings can be useful to inform treatment decisions.

**Electronic supplementary material:**

The online version of this article (doi:10.1186/s12890-015-0034-y) contains supplementary material, which is available to authorized users.

## Background

The emergence of new evidence assessing the effectiveness of therapies for idiopathic pulmonary fibrosis (IPF) has created considerable interest and hope amongst patients, carers and clinicians. IPF is a devastating lung disease characterised by the deposition of excessive scar tissue within the lungs, which leads to breathlessness and ultimately respiratory failure and death. It has been well documented that the incidence of IPF is increasing but the reasons for this are unclear. High mortality rates are also well reported, and based on reported 5-year survival rates these would place IPF seventh on a list of fatal malignancies [1,2]. Until recently few treatment options have been available, so the focus of treatment for many patients has been symptom control and palliation [3].

The new optimism has stemmed from the fact pirfenidone, licensed in Europe in 2011 on the basis of evidence from 4 randomised controlled trials (RCTs) [4-6], has been shown in a recent multicentre RCT to slow the rate of progression of IPF [7]. Furthermore another agent (nintedanib) also met its primary end-point of reducing the annual rate of pulmonary function decline in two concurrent RCTs [8]. On the strength of these data the Food and Drug Administration (FDA) and the European Medicine Agency have recently approved both drugs for use in IPF. These treatments have the potential for offering new hope for patients and carers [9] and clinicians will be eager to offer patients the most appropriate treatment. It is therefore imperative to consider the weight of all the evidence for each of these treatments.

The purpose of this study was to systematically review the clinical effectiveness of treatments for IPF and present the findings of a network meta-analysis (NMA) of key outcomes. We followed the principles of the Preferred Reporting Items for Systematic Reviews and Meta-Analyses (PRISMA) Statement [10].

## Methods

The methods for this systematic review are described in a research protocol which is registered with the International Prospective Register of Systematic Reviews [11]. This is an update of a previous systematic review which also considered cost-effectiveness [12]. We identified articles by searches of MEDLINE, EMBASE and the Cochrane Library from database inception until May 2014 (for search strategies see Additional file 1: Table S1). No language restrictions were applied. Two independent reviewers screened titles and abstracts for inclusion and full text articles were retrieved for further scrutiny. These were reviewed by one reviewer and checked by a second to identify RCTs that included participants with a confirmed diagnosis of IPF. Eligible interventions were N-acetylcysteine (NAC) alone or in combination, pirfenidone or nintedanib, assessed on outcomes measuring indices of lung function/capacity, exercise performance, quality of life and adverse events.

Data extracted from studies included participant and study characteristics, intervention and comparator details and results. An assessment of the methodological quality of each included study was made [13]. Only published data were included. One reviewer undertook data extraction and quality assessment. These were checked by a second reviewer and differences in opinion were resolved through discussion with a third reviewer. We synthesised data in a narrative review and a Bayesian NMA [14,15]. In circumstances where randomised evidence between all relevant comparators is unavailable, network meta-analysis combines evidence from trials comparing different sets of treatments that form a connected evidence network through common comparators, in this case placebo. It retains within trial randomisation, allowing direct and indirect evidence to inform estimates of relative treatment effect in a single analysis. A vague prior distribution (a normal distribution with mean zero and variance 10,000) that contains little information relative to the likelihood was used to ensure that results were based on the data and not the choice of prior [16].

The NMA assessed five endpoints from 11 studies. An advantage of NMA is that where interventions have not been directly compared in RCTs the results of different trials may be used to estimate the relative treatment effect between two treatments through indirect comparison. In addition, the analysis allows for meta-regression to control for study level covariates which may be a source of heterogeneity between trials [17].

The decline in FVC may be measured in two ways: as a decline in FVC % predicted; and as an absolute change from baseline (litres). In addition, some trials report vital capacity, which can be assumed to be the same as FVC in IPF. We used the standardised mean difference (SMD) approach to convert these measurements to a common scale using Hedges’ adjusted g method to overcome potential small sample bias [18]. The SMDs were then converted to log odds ratios (logOR) using the method of Chinn [19] to facilitate interpretation.

Our base case used the FVC % predicted where reported, as by definition this includes adjustment for age and sex. We conducted sensitivity analyses using litres to examine variation in results arising from the different measurement scales, and controlling for baseline FVC through meta-regression.

The NMA was performed in WinBUGS using code adapted from Dias and colleagues [15], see Additional file 1: Appendix. Two chains were run for 50,000 simulations with a burn-in period of 20,000 and a thinning interval of 2, giving a final sample size of 30,000. Trace, Brooks-Gelman-Rubin, and density plots were examined to establish model convergence [20]. Fixed and random effects models were conducted with best model fit determined by the deviance information criterion [21].

## Results

**Figure 1:**
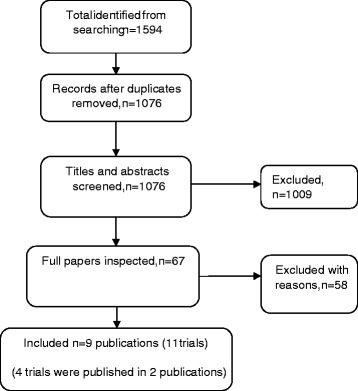
PRISMA flow-chart of included studies.

Searches yielded 1076 unique references and 67 of these were retrieved after initial screening. Of these we included 11 studies (Figure 1). Five RCTs evaluated the use of pirfenidone [4-7], three NAC [22-24], and three nintedanib. [8,25] Two of the NAC studies [22,23] are in fact one three arm RCT and as such there is not complete independence in the placebo arms. Table 1 provides summary descriptions of each study. The participants in all studies would likely be classed as mild to moderate IPF, with baseline FVC [26] ranging from approximately 68% to 89% of predicted values. Participants were around 64–68 years old, and were predominantly men. In most studies diagnosis was within the previous two years. The ratio of the forced expiratory volume in the first second to the FVC was not reported consistently across the studies. In three recent RCTs of pirfenidone [7] and nintedanib [7,8] this was required to be at least 0.80 and at least 0.70 for study inclusion respectively. For the RCT of NAC [22,23] this was required to be at least 0.65. Although there were subtle differences in trial inclusion criteria and diagnostic criteria, baseline characteristics were broadly similar and are deemed to be generally reflective of IPF patients with mild-to-moderate disease seen in clinical practice. As such we judged these populations to be similar enough to be combined in meta-analysis. Primary outcomes were based on the FVC in all but one trial [6]. With one exception [24], the studies had reasonable sample sizes and were adequately powered to test differences in their primary outcomes. Treatment duration ranged between eight and 16 months, with the majority following up until approximately 12 months.

**Table 1:** **Characteristics of randomised controlled trials included in the meta-analysis**

**Source**	**No. of participants (Intervention)**	**Duration treatment, wk**	**Age, Mean**	**Male %**	**Time since diagnosis, yr**	**FVC, %, mean**	**Risk of bias** ^**a**^
Noble et al. 2011 [4] (Capacity 06)	171 (Pirfenidone 2403 mg/day)	72	67	72	≤1 yr: 59%	74	Low
	173 (Placebo)						
Noble et al. 2011 [4] (Capacity 04)	174 (Pirfenidone 2403 mg/day)	72	66	71	≤1 yr: 48%	75	Low
	174 (Placebo)						
Taniguchi et al. 2010 [5]	108 (Pirfenidone 1800 mg/day)	52	65	78	<1 yr: 37%	78	Unclear
	104 (Placebo)						
Azuma et al. 2005 [6]	73 (Pirfenidone 1800 mg/day)	39	64	90	<1 yr: 22%	80	Unclear
	36 (Placebo)						
King et al. 2014 [7] (Ascend)	278 (Pirfenidone 2403 mg/day)	52	68	78	1.7	68	Low
	277 (Placebo)						
Richeldi et al. 2011 [25]	85 (Nintedanib 300 mg/day)	52	65	75	1.2	80	Low
	85 (Placebo)						
Richeldi et al. 2014 [8] (INPULSIS-1)	309 (Nintedanib 300 mg/day)	52	67	81	1.7	80	Low
	204 (Placebo)						
Richeldi et al. 2014 [8] (INPULSIS-2)	329 (Nintedanib 300 mg/day)	52	67	78	1.6	79	Low
	219 (Placebo)						
Homma et al. 2012 [24],	38 (Inhaled NAC)	48	68	76	3	89	Unclear
	38 (Placebo)						
Raghu et al. 2012 [22], (PANTHER)	77 (NAC triple therapy)	32	68	75	1.1	71	Low
	78 (Placebo)						
IPFCRN, 2014 [23] (PANTHER)	133 (NAC)	60	68	78	1.1	73	Low
	131 (Placebo)						

^a^Risk of selection bias.

Overall the studies were assessed as having a low or uncertain risk of bias based on the assessment of adequacy of concealment of allocations prior to assignment to groups. All but one trial [24] were assessed as having adequate randomisation procedures, and blinding was at an unclear or low risk of bias in all but one trial [24]. Most trials described using an intention to treat (ITT) analysis, although not all reported details. Several trials presented adjusted analyses incorporating a range of covariates and interactions [4,5,7,8,22,23]. As the adjustments in the analyses were to take account of within-study variability in the outcome associated with the covariates, it did not negate meta-analysis of studies with and without adjustment.

### FVC

**Figure 2:**
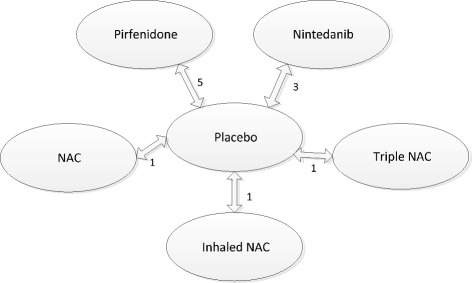
Evidence network for forced vital capacity endpoint. Numbers on arrows refer to number of studies informing the comparison.

**Figure 3:**
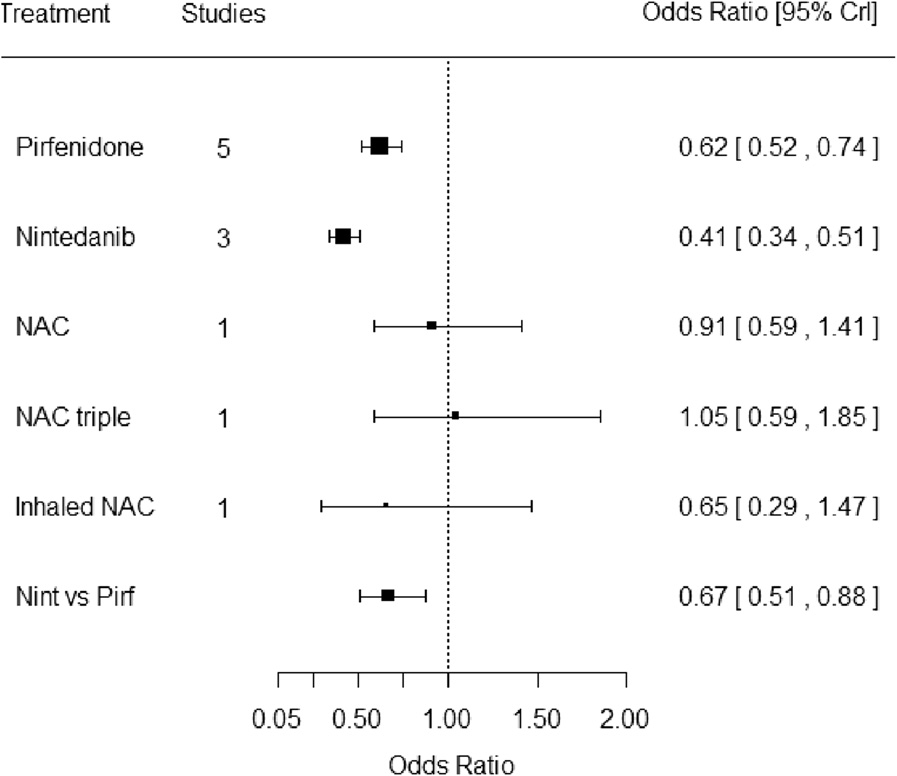
Results of the network meta-analysis for forced vital capacity.

FVC outcome data used in the NMA can be seen in Table 2 and the evidence network in Figure 2. Trials were relatively homogeneous which validates the use of fixed effect models. When combining the different FVC treatment effects only pirfenidone and nintedanib produce a statistically significant slowing in the rate of FVC decline compared with placebo (ORs < 1 with 95% credible intervals [Crl] all less than 1) (Figure 3). These two treatments were compared indirectly using the placebo as the common comparator, and results indicate that nintedanib is statistically significantly better than pirfenidone in slowing FVC decline (OR 0.67, 95% CrI 0.51, 0.88). Random effects analysis of FVC supports the trends seen although the results were not statistically significant. Sensitivity analysis controlling for baseline FVC had little impact on results. Using FVC decline in litres, where reported, also gave similar results.

**Table 2:** **Forced vital capacity outcomes reported in the included trials**

	**Change in percent predicted FVC (%) or absolute change from baseline (L)**						**No. with reduction in FVC >10%** ^**a b**^		
**Source**	**FVC outcome**	**Treatment**		**Placebo**		**P value**	**Treatment**	**Placebo**	**P value**
		**No. of participants**	**Mean (SD)**	**No. of participants**	**Mean (SD)**		No. (%)	No. (%)	
**Pirfenidone**									
Noble et al. 2011 [4] (Capacity 006)	% pred	171	−9.0 (19.6)	173	−9.6 (19.1)	0.501	39 (23)	46 (27)	0.440
Noble et al. 2011 [4] (Capacity 004)	% pred	174	−8.0 (16.5)	174	−12.4 (18.5)	0.001	35 (20)	60 (35)	0.01
Taniguchi et al. 2010 [5]	Litres	104	−0.09 (0.20)	103	−0.16 (0.20)	0.042			
Azuma et al. 2005 [6]	Litres	72	−0.03 (0.22)	35	−0.13 (0.19)	0.037	9 (13)	12 (36)	0.003
King et al. 2014 [7] (Ascend)	Litres	278	−0.122 (0.4)	277	−0.262 (0.4)	0.001	46 (16.5)	88 (31.8)	<0.00001
**Nintedanib**									
Richeldi et al. 2011 [25]	% pred	85	−1.04 (9.1)	85	−6.0 (9.4)	<0.001	20 (23.8)	37 (44.0)	0.004
	Litres	85	−0.06 (0.37)	85	−0.23 (0.37)	0.001			
Richeldi et al. 2014 [8] (INPULSIS-1)	% pred	307	−2.8 (6.2)	204	−6.0 (6.2)	<0.001	91 (29.5)^c^	88 (43.1)	<0.001
	Litres	307	−0.095 (0.22)	204	−0.205 (0.22)	<0.001			
Richeldi et al. 2014 [8] (INPULSIS-2)	% pred	327	−3.1 (6.99)	217	−6.2 (6.99)	<0.001	100 (30.4)^e^	79 (36.1)^e^	0.18
	Litres	327	−0.095 (0.23)	217	−0.205 (0.23)	<0.001			
**Inhaled NAC**									
Homma et al. 2012 [24],	Litres	38	−0.09 (0.3)	38	−0.15 (0.2)	0.27			
**NAC triple therapy**									
Raghu et al. 2012 [22], (PANTHER)	Litres	77	−0.24 (0.4)	78	−0.23 (0.4)	0.85			
**NAC**									
IPFCRN, 2014 [23] (PANTHER)	% Pred ^d^	133	−4.37 (7.5)	131	−4.76 (7.3)	0.67			
	Litres ^d^	133	−0.18 (0.3)	131	−0.19 (0.3)	0.77			

In some trials the vital capacity was reported which has been assumed to translate to the FVC.

Where not reported in individual trials measures of variance have been estimated from p-values reported using standard methodology.

^a^Number of participants with a reduction in mean FVC of >10% (or 200 ml where applicable). ^b^Number of participants with a reduction in mean FVC of >10% or death in ASCEND trial [7]. ^c^Based on 309 patients in Nintedanib group. ^d^From longitudinal analysis which adjusts for treatment time, interaction between time and treatment, age, sex, race and height; assumes that data were missing at random and no data were imputed. ^e^Based on 329 patients in Nintedanib group and 219 patients in placebo group.

A number of trials reported FVC as a dichotomous outcome, using the proportion with a decline in FVC % predicted of 10% or more (see Table 2). In our NMA both treatments are associated with statistically significantly lower odds of a decline in FVC % predicted of ≥10% compared to placebo. The indirect comparison of nintedanib and pirfenidone indicates that pirfenidone is associated with slightly lower odds of a decline in FVC % predicted of ≥10% compared to nintedanib, but that this is non-significant (OR 1.21, 95% CrI 0.86, 1.72), Additional file 1: Figure S1. Although not significant this trend is divergent to the other FVC outcomes and may be related to differences in definitions in the studies. For example in one trial [7] this was the proportion with a reduction >10% in FVC or death. It is also uncertain whether studies reported this 10% decline as an absolute or a relative decline. Three trials reported results as an annualised decline in FVC but the network for this outcome was not complete for comparison.

The FVC treatment effect versus placebo in one pirfenidone trial [7] appears to be large when compared with other trials. Two scenario analyses, first to exclude this trial from the FVC % predicted model, and second to adjust the numbers with an FVC decline greater than 10% to remove all cause deaths, did not substantively alter the outputs of the NMA.

### Acute exacerbations

Evidence of acute exacerbations were reported in some of the included trials but there were some differences in rates reported. One trial [25] only reported the incidence per 100 years which we converted using the study sample size to rate per year, and two trials [8] reported the proportion of participants with at least one event (Additional file 1: Table S2). Acute exacerbations are relatively rare occurrences and studies were not powered to detect a difference in these rates, therefore results should be interpreted cautiously (for the OR for acute exacerbation versus placebo, see Additional file 1: Figure S2). Both nintedanib and pirfenidone have favourable point estimates, however only the OR for nintedanib achieves significance. Owing to a number of uncertainties with these data no indirect comparison was undertaken.

### Mortality

All-cause mortality and respiratory-related mortality were reported in trials of all treatments with the exception of inhaled NAC (Additional file 1: Table S3). The NMA ORs for all-cause mortality compared with placebo showed that both nintedanib and pirfenidone had favourable point estimates, however, only pirfenidone was statistically significant (Additional file 1: Figure S3). In indirect comparison pirfenidone is associated with lower odds of all-cause mortality compared to nintedanib, but this is not significant (OR 1.39, 95% CrI 0.70, 2.82). Similar results were seen for respiratory mortality, see Additional file 1: Table S4 and Figure S4. Studies were not powered to detect a difference on mortality and therefore the results should be considered cautiously.

### Other outcomes

The included trials reported other outcomes less consistently and are therefore not appropriate for meta-analysis. No studies that reported it showed significant effects of treatment on diffusing capacity of the lung [4-6,22,23], and effects on the six-minute walk test were mixed across the trials with only three showing a positive effect [4,7,22]. Few studies reported dyspnoea and results were not significant [4,22,23]. On the St George’s Respiratory Questionnaire, a measure of quality of life, there were mixed results but overall it would appear that the treatments have little effect on quality of life.

## Discussion

This study presents a comprehensive review of evidence of the effectiveness of three treatments for IPF and is a timely addition to the ongoing appraisal of recently published trials by patients and clinicians. Evidence for FVC shows a significantly slower decline in patients treated with pirfenidone or nintedanib compared to placebo. When compared indirectly, there was a slower decline in FVC for those treated with nintedanib than for pirfenidone. On endpoints thought more important for patients, such as mortality and acute exacerbations, there were mixed results. Nintedanib was significantly better than placebo for acute exacerbations and pirfenidone was significantly better than placebo for mortality and respiratory mortality. Indirect comparisons of mortality outcomes were not statistically significant.

Some aspects of the evidence included in the review should be taken into account when interpreting the findings. The study is limited by inconsistent reporting of the key outcomes in the trial publications and definitions of outcomes differed. It has been questioned whether the participants in one RCT (ASCEND) [7] differ from other trials [9]. Although our assessment of heterogeneity suggests no statistically significant heterogeneity, the rate of decline in FVC in the placebo group appears to tail off near to the end of the trial. On observation of placebo arms of all other included trials this is at a greater rate, although the slope of decline seen in the more recently published trials are more similar. One possible reason for this reduction may be related to the effects of the imputation of FVC data for patients who died, however, in scenario analyses to test the consistency of results we would suggest that the difference between groups is similar to the other pirfenidone trials. Finally, four of the trials did not report ITT analysis, which is often recommended as the least biased way to estimate intervention effects in RCTs [27]. It is possible therefore that treatment effects may be exaggerated in these trials [5,6,8,24].

On FVC, within individual trials, results have not been entirely consistent in their comparisons with placebo. However, through NMA results show statistically significant effects for both of pirfenidone and nintedanib. Both treatments have received authorisation from the FDA and in Europe. It is anticipated that the findings of our indirect comparisons between nintedanib and pirfenidone will be of use to inform decision making in the clinical setting. This is particularly important given that until recently there has been an unmet need. Few trials reported estimates of quality of life and good quality research to establish the quality of life of patients with IPF should be a priority. This would allow a fuller assessment of the effectiveness of these treatments. Additionally, these treatments do not stabilize or reverse the decline in IPF and more research into treatments with the potential to meet this aim is required.

Our review and NMA has been undertaken following recognised principles for undertaking a systematic review to ensure that our analyses are transparent and as unbiased as possible. Additionally we ensured that only the highest quality studies were included to limit uncertainty in the results. Our group has no direct vested interest in the pivotal trials. Limitations to our study are that the NMA for FVC assumes the different measures of FVC are equivalent and we converted results to odds ratios to provide meaningful results. Although recognised methodologies were used, these factors should be considered when interpreting the results. Assumptions of homogeneity, similarity, and consistency between direct and indirect evidence in our NMA were made [28], however, uncertainties remain. For example, there could be other sources of observed or unobserved heterogeneity which could impact on relative treatment effects.

## Conclusions

Beneficial treatment effects were demonstrated across key outcomes for two included interventions. In indirect comparison nintedanib was associated with significantly better outcome on slowing the decline in FVC than pirfenidone and this finding was robust in sensitivity analyses. Mortality rates showed trends in favour of pirfenidone but these were not statistically significant. Our findings can be used to help inform treatment decisions for this population of patients who following the recent regulator’s approvals will benefit from greater access to these therapies.

## Additional file

Additional file 1: Table S1. MEDLINE search strategy. **Appendix.** NMA Model code. **Figure S1.** FVC categorical (>10% decline). **Table S2.** Acute exacerbations reported in the included trials. **Figure S2.** Acute exacerbations. **Table S3.** All-cause mortality. **Figure S3.** All-cause mortality. **Table S4.** Respiratory mortality. **Figure S4.** Respiratory mortality.

## Abbreviations

CrI: Credible interval
FVC: Forced vital capacity
IPF: Idiopathic pulmonary fibrosis
ITT: Intent-To-Treat
NAC: N-acetylcysteine
NMA: Network meta-analysis
OR: Odds ratio
RCT: Randomised controlled trial
SD: Standard deviation
SMD: Standardised mean difference
VC: Vital capacity
