# Supplementary material for: Comparing new treatments for idiopathic pulmonary fibrosis – a network meta-analysis
Source: BMC Pulm Med. 2015 Apr 18;15:37. doi: 10.1186/s12890-015-0034-y (PMC4429373; doi:10.1186/s12890-015-0034-y)
Supplement: Additional file 1: Table S1. — MEDLINE search strategy. Appendix. NMA Model code. Figure S1. FVC categorical (>10% decline). Table S2. Acute exacerbations reported in the included trials. Figure S2. Acute exacerbations. Table S3. All-cause mortality. Figure S3. All-cause mortality. Table S4. Respiratory mortality. Figure S4. Respiratory mortality. [file 12890_2015_34_MOESM1_ESM.docx]

**Online supplementary tables and figures**

**e-Table 1: MEDLINE search strategy**

| The MEDLINE search strategy (presented below) for the systematic review of clinical effectiveness was adjusted as necessary for other electronic databases. Search strategies for the systematic review are available from the authors on request. |
| --- |
| 1 idiopathic pulmonary fibrosis/ (548)  2 IPF.tw. (1925)  3 (idiopath$ and pulmonary and fibro$).tw. (3334)  4 (idiopath$ and (lung and fibro$)).tw. (2502)  5 (((usual or ordinary) adj3 interstiti$) and pneumo$).tw. (698)  6 (((nonspecific or "non specific") adj3 interstitial) and pneumo$).tw. (583)  7 (idiopath$ and interstiti$ and pneumoni$).tw. (1383)  8 ("usual interstiti$" adj5 (lung or pulmonary or alveoli$)).tw. (191)  9 ("nonspecific interstiti$" adj5 (lung or pulmonary or alveoli$)).tw. (83)  10 ("non specific interstiti$" adj5 (lung or pulmonary or alveoli$)).tw. (38)  11 (cryptog$ and fibro$ and alveoli$).tw. (327)  12 or/1-11 (5331)  13 ("lung disease$" adj5 (interstiti$ or fibrosis or fibrotic)).tw. (5421)  14 pulmonary fibrosis/ (15120)  15 exp lung diseases interstitial/ (42412)  16 (pulmonary adj5 (fibrosis or fibrotic)).tw. (10738)  17 (interstiti$ adj5 (pneumonia or lung or pulmonary or alveoli$)).tw. (12889)  18 "diffuse parenchymal lung disease".tw. (82)  19 or/13-18 (65328)  20 (idiopathic or unexplained or nonspecific or "non specific").tw. (178936)  21 (((unknow$ or uncertain$) adj4 (origin$ or cause$ or aetiol$ or etiol*)) or idiopa$).tw. (102573)  22 20 or 21 (207520)  23 19 and 22 (6196)  24 12 or 23 (7352)  25 (cystic adj fibro$).mp. (33749)  26 24 not 25 (7191)  27 limit 26 to humans (6808)  28 limit 26 to animals (785)  29 26 not 27 not 28 (81)  30 27 or 29 (6889)  31 Randomized Controlled Trials as Topic/ (77921)  32 randomized controlled trial.pt. (322018)  33 controlled clinical trial.pt. (83725)  34 Controlled Clinical Trial/ (83725)  35 placebos/ (30626)  36 random allocation/ (73596)  37 Double-Blind Method/ (113512)  38 Single-Blind Method/ (15853)  39 (random* adj2 allocat*).tw. (17050)  40 placebo*.tw. (133778)  41 ((singl* or doubl* or trebl* or tripl*) adj (blind* or mask*)).tw. (111089)  42 crossover studies/ (29092)  43 (crossover* or (cross adj over*)).tw. (49865)  44 Research Design/ (65242)  45 ((random* or control*) adj5 (trial* or stud*)).tw. (424830)  46 Clinical Trials as Topic/ (158570)  47 trial.ti. (97491)  48 randomly.ab. (164073)  49 (randomized or randomised).ab. (271090)  50 or/31-49 (1044973)  51 3 and 50 (257)  52 limit 30 to (controlled clinical trial or randomized controlled trial) (102)  53 51 or 52 (292)  54 limit 30 to meta analysis (7)  55 53 or 54 (294)  56 (rat or rats).ti. (676338)  57 55 not 56 (292)  58 (official and "idiopathic pulmonary fibrosis").ti. (1)  59 57 OR 58 (293) |

**e-Appendix: NMA Model code**

WinBUGs code (adapted from DSU report 2)(Dias et al, 2012)

# Fixed effects model for two-arm trials

model{ # *** PROGRAM STARTS

for(ii in 1:ns) { # LOOP THROUGH 2-ARM STUDIES

y[ii,2] ~ dnorm(md[ii,2],prec[ii,2]) # normal likelihood for 2-arm trials

var[ii,2] <- pow(se[ii,2],2) # calculate variances

prec[ii,2] <- 1/var[ii,2] # set precisions

dev[ii,2] <- (y[ii,2]-md[i,2])*(y[ii,2]-md[ii,2])*prec[ii,2] #Deviance contribution

md[ii,2] <- d[t[ii,1]] - d[t[ii,2]] # mean of treat effects distributions

}

totresdev <- sum(dev[,2]) #Total Residual Deviance

d[1]<-0 # treatment effect is zero for reference treatment

for (kk in 2:nt){ d[kk] ~ dnorm(0,.0001) } # vague priors for treatment effects

} # *** PROGRAM ENDS

# Data (IPF – trial-level data: standardised treatment differences)

list(ns=10, nt=8)

t[,1] t[,2] y[,2] se[,2]

2 1 0.234793937 0.461455736 #Raghu 1991

3 1 0.457405244 0.155432826 #Richeldi 2011

4 5 0.343971125 0.170963162 #Demedts 2005

4 1 -0.02487725 0.160653585 #IPFCRN 2012

6 1 0.232946081 0.230234875 #Homma 2012

7 1 0.471090122 0.208655219 #Azuma 2005

7 1 0.250474568 0.107635613 #Capacity 004

7 1 0.030939458 0.107841122 #Capacity 006

7 1 0.348717949 0.140084088 #Taniguchi 2010

8 1 0.065705107 0.149121516 #IPFCRN 2010

END

# 1=placebo

# 2= Azathioprine

# 3=BIBF 1120

# 4=NAC triple therapy

# 5=Azathioprine+placebo

# 6=Inhaled NAC

# 7=Pirfenidone

# 8=Sildenafil

# Initial Values

#chain 1

list(d=c(NA, 0,0,0,0, 0,0,0))

#chain 2

list(d=c(NA, 1,1,1,1, 1,1,1))


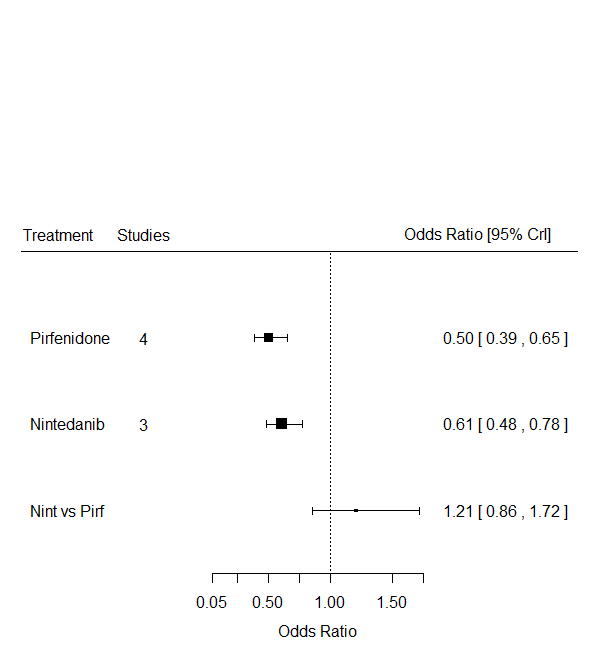


**e-Figure 1: FVC categorical (>10% decline)**

**e-Table 2– Acute exacerbations reported in the included trials**

| **Source** | **Outcome** | **Treatment** | | **Placebo** | | **P value** |
| --- | --- | --- | --- | --- | --- | --- |
|  |  | **No. of Participants** | **No. (%)** | **No. of Participants** | **No. (%)** |  |
| **Pirfenidone** |  |  |  |  |  |  |
| Noble et al, 2011^20^ (Capacity 006) |  | 171 |  | 173 |  |  |
| Noble et al, 2011^20^ (Capacity 004) |  | 174 |  | 174 |  |  |
| Taniguchi et al, 2010^21^ |  | 108 | 6 (5.6) | 104 | 5 (4.8) | ns |
| Azuma et al, 2005^22^ |  | 72 | 0 | 35 | 5 (14) | 0.0031 |
| King et al, 2014^4^ (Ascend) |  |  |  |  |  |  |
| **Nintedanib** |  |  |  |  |  |  |
| Richeldi et al, 2011^26^ | Incidence of acute exacerbations, n per 100 patient years | 85 | 2.4 | 85 | 15.7 | 0.02 |
| Richeldi et al, 2014^5^ (INPULSIS-1) ^a^ | N (%) with at least one acute exacerbation.  [Incidence of acute exacerbations per 100 patient years] | 309 | 19 (6.1)  [6.6] | 204 | 11 (5.4)  [5.6] | 0.68 |
| Richeldi et al, 2014^5^ (INPULSIS-2) ^a^ | N (%) with at least one acute exacerbation.  [Incidence of acute exacerbations per 100 patient years] | 329 | 12 (3.6)  [3.9] | 219 | 21 (9.6)  [10.2] | 0.007 |
| **Inhaled NAC** |  |  |  |  |  |  |
| Homma et al 2012,^25^ |  | 38 |  | 38 |  |  |
| **NAC triple therapy** |  |  |  |  |  |  |
| Raghu et al 2012,^23^  (PANTHER) | Acute exacerbation rate | 77 | 5 (6) | 78 | 0 | nr |
| **NAC** |  |  |  |  |  |  |
| IPFCRN, 2014^24^  (PANTHER) | Acute exacerbations | 133 | 3 (2.3) | 131 | 3 (2.3) | >0.99 |

**^a^** Time to first exacerbation also reported.

**
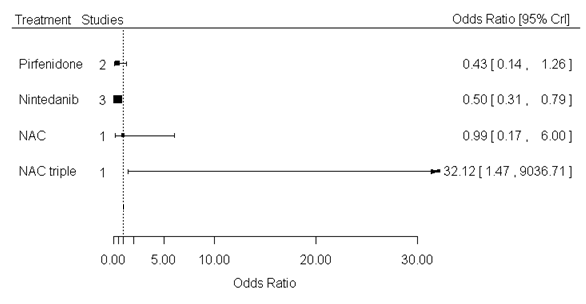
**

**e-Figure 2: Acute exacerbations**

A continuity correction of 0.5 was added to the incidence of acute exacerbations in the placebo arm for triple NAC as this is a zero value.

**e-Table 3: All-cause mortality**

|  | **Mortality** |  | |  | |  |
| --- | --- | --- | --- | --- | --- | --- |
| **Source** | **Outcome** | **Treatment** | | **Placebo** | | **P value** |
|  |  | **No. of Participants** | **No. (%)** | **No. of Participants** | **No. (%)** |  |
| **Pirfenidone** |  |  |  |  |  |  |
| Noble et al, 2011^20, 30^ (Capacity 006) |  | 171 | 6 (3.5) | 173 | 9 (5.2) | 0.44 |
| Noble et al, 2011^20, 30^ (Capacity 004) |  | 174 | 5 (2.9) | 174 | 13 (7.5) | 0.05 |
| Taniguchi et al, 2010^21^ |  |  |  |  |  |  |
| Azuma et al, 2005^22^ |  |  |  |  |  |  |
| King et al, 2014^4^ (Ascend) |  | 278 | 11 (4) | 277 | 20 (7.2) | 0.1 |
| **Nintedanib** |  |  |  |  |  |  |
| Richeldi et al, 2011^26^ |  | 85 | 7 (8.2) | 85 | 9 (10.6) |  |
| Richeldi et al, 2014^5^ (INPULSIS-1 and 2 pooled data) |  | 638 | 35 (5.5) | 423 | 33 (7.8) | 0.14 |
| **Inhaled NAC** |  |  |  |  |  |  |
| Homma et al 2012,^25^ |  |  |  |  |  |  |
| **NAC triple therapy** |  |  |  |  |  |  |
| Raghu et al, 2012^23^  (PANTHER) |  | 77 | 8 (10) | 78 | 1 (1) |  |
| **NAC** |  |  |  |  |  |  |
| IPFCRN, 2014^24^  (PANTHER) |  | 133 | 6 (4.9)^a^ | 131 | 3 (2.5)^a^ | 0.3 |

^a^ from Kaplan Meier analysis over 60 week study period; also reported as 6/133 (4.5%) versus 3/131 (2.3%) p=0.5.


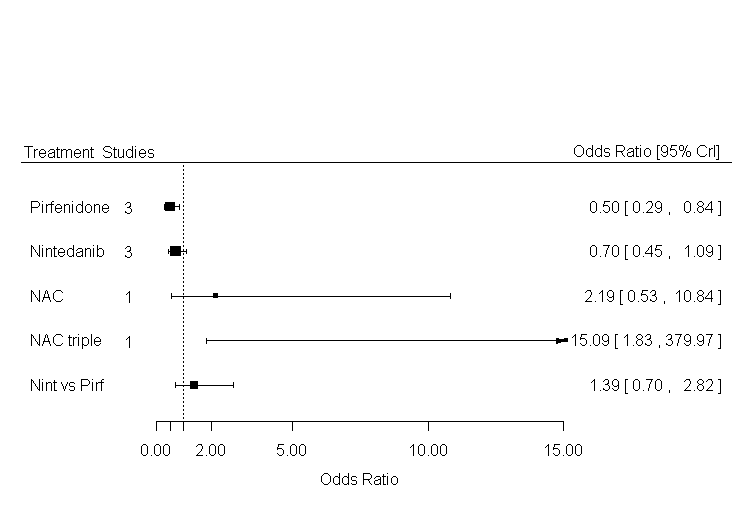


**e-Figure 3: All-cause mortality**

**e-Table 4: Respiratory mortality**

| **Source** | **Outcome** | **Treatment** | | **Placebo** | | **P value** |
| --- | --- | --- | --- | --- | --- | --- |
|  |  | **No. of Participants** | **No. (%)** | **No. of Participants** | **No. (%)** |  |
| **Pirfenidone** |  |  |  |  |  |  |
| Noble et al, 2011^20, 30^ (Capacity 006) |  | 171 | 2 (1.2) | 173 | 7 (4.0) | 0.1 |
| Noble et al, 2011^20, 30^ (Capacity 004) |  | 174 | 2 (1.1) | 174 | 8 (4.6) | 0.06 |
| Taniguchi et al, 2010^21^ |  |  |  |  |  |  |
| Azuma et al, 2005^22^ |  |  |  |  |  |  |
| King et al, 2014^4^ (Ascend) |  | 278 | 3 (1.1) | 277 | 7 (2.5) | 0.23 |
| **Nintedanib** |  |  |  |  |  |  |
| Richeldi et al, 2011^26^ |  | 85 | 2 (2.4) | 85 | 8 (9.4) | 0.06 |
| Richeldi et al, 2014^5^ (INPULSIS-1 and 2 pooled data) |  | 638 | 24 (3.8) | 423 | 21 (5.0) | 0.34 |
| **Inhaled NAC** |  |  |  |  |  |  |
| Homma et al 2012,^25^ |  |  |  |  |  |  |
| **NAC triple therapy** |  |  |  |  |  |  |
| Raghu et al, 2012^23^  (PANTHER) |  | 77 | 7 (9) | 78 | 1 (1) | 0.02 |
| **NAC** |  |  |  |  |  |  |
| IPFCRN, 2014^24^  (PANTHER) |  | 133 | 5 (3.8) | 131 | 3 (2.3) | 0.72 |


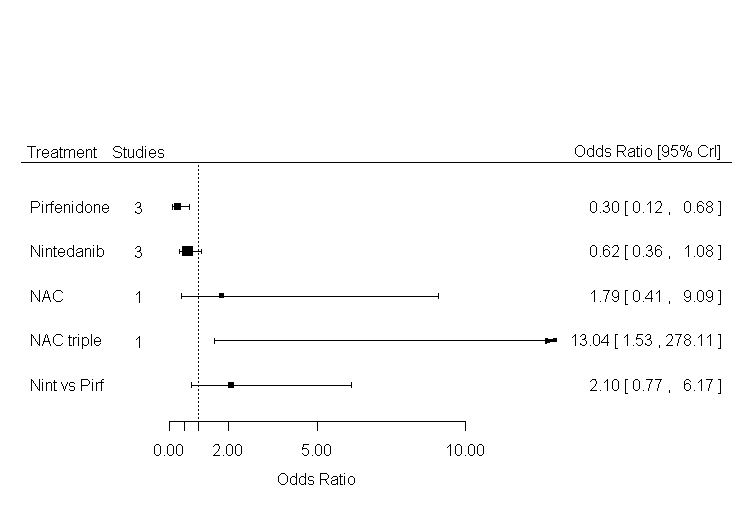


**e-Figure 4: Respiratory mortality**
